# Fatigue and Physical Activity in People after COVID-19 in Poland

**DOI:** 10.3390/jpm13091369

**Published:** 2023-09-11

**Authors:** Anna Zalewska, Monika Gałczyk

**Affiliations:** Faculty of Health Sciences, University of Lomza, 14 Akademicka St., 18-400 Lomza, Poland; monikagalczyk@onet.eu

**Keywords:** post COVID, fatigue, physical activity, adults, MFIS, IPAQ

## Abstract

Objectives: The purpose of this research was to look at the amount of fatigue and physical activity (PA) in individuals after COVID-19 in Poland and the correlation between fatigue and PA. Methods: The online research was carried out among adult Polish residents (122 women and 82 men) who had tested positive for SARS-CoV-2 during the previous year. The level of fatigue was measured using the Modified Fatigue Impact Scale (MFIS). The PA level was assessed using the International Physical Activity Questionnaire (IPAQ). Results: A total of 46.6% of the subjects had been ill with COVID-19 for more than 6 months before the time of the survey response. The MFIS total measure is 77 of the maximum score, and the median is 17. A total of 26% of respondents reported low activity, while 41% of respondents reported high activity. A statistically significant negative relationship was found between PA level and total fatigue score. The best scores for fatigue and PA were obtained by the subjects with mild COVID-19. The time since diagnosis (as opposed to older age and female sex) was not clearly associated with most measures. Conclusions: PA may play an important role in regulating the severity of fatigue; it should be increased, especially in patients after COVID. Further studies are also needed to investigate the mechanism of differences in fatigue and PA.

## 1. Introduction

COVID-19 has been shown to have negative effects on patients’ psychophysical health, including an increase in fatigue and a decrease in overall physical performance [1,2,3]. The terms “long COVID” or “post COVID” refer to symptoms that occur after SARS-CoV-2 infection and last ≥12 weeks with no other diagnosis and no predictable time of resolution. The recovery of a patient with COVID-19 depends on a number of variables, such as multiple disorders, the intensity of the COVID-19 infection, and age. Some symptoms, such as fatigue, may persist for weeks [4]. At the same time, fatigue is one of the most frequently reported chronic problems among individuals who have previously been infected with SARS-CoV-2 [5]. The phenomenon is still poorly understood, and there is no causal mechanism [6]. Post COVID-19 fatigue is usually defined as a decrease in physical or mental (or physical and mental) activity, which may be due to changes in various factors, e.g., peripheral, central, and psychological, originating in COVID-19 disease [5]. In addition, fatigue is treated as an unpleasant subjective symptom that involves sensations that emanate from the whole body and impede the individual’s capacity to function in daily life [6].

Fatigue predicts a decrease in physical activity (PA) and performance [7]. The relationship between the level of fatigue and the level of PA is multifactorial. It can be clarified by body composition, physical fitness, or concomitant diseases, among other factors [8]. At the same time, many factors can influence fatigue and PA levels after SARS-CoV-2 virus infection.

A sufficient level of PA is an essential factor in maintaining health, especially after COVID-19 [9]. The beneficial effects of PA on physical and mental wellbeing have long been known. Regular exercise improves mood, reduces stress and, most importantly, reduces the risk of numerous persistent diseases, such as cardiovascular failure, cancer, and depression [10,11,12].

Even before the COVID-19 pandemic, 2/3 of adult women and men in Poland did not reach the level of PA suggested by specialists [13]. Longitudinal studies conducted in recent years have confirmed that the levels of PA declined sharply during and after the relaxation of pandemic restrictions [14,15].

These declines have also been observed in individuals suffering from chronic conditions, such as obesity and hypertension [16]. PA at an appropriate level is one of the pillars of a healthy lifestyle [17]. Even before the COVID-19 pandemic, it was clear that moderate PA reduces the duration, severity, and frequency of upper respiratory tract infections [18], and regular exercise also reduces the morbidity and mortality of influenza and pneumonia [19]. A study among SARS-CoV-2 positive adults conducted during the pandemic confirmed that those who engaged in the recommended level of PA were less likely to be infected with SARS-CoV-2 and less likely to suffer from severe COVID-19 and the associated risk of death. This suggests that maintaining appropriate levels of PA has significant health value and has been shown to have measurable benefits against COVID-19 [20].

Researchers and clinicians meeting at the Virtual Meeting of the Physiological Society emphasised the importance of monitoring levels of PA and fatigue in the population, among other factors [21].

The aim of this research was to investigate the degree of fatigue and PA as well as the connection between fatigue and PA in people after COVID-19 in Poland. The authors additionally posed the following research question: Is there an association between the type of COVID-19 transmission, time since diagnosis, gender, age, and level of fatigue and PA in people after COVID-19 in Poland?

## 2. Material and Methods

The detailed methodology and baseline characteristics of the study have been described in a previous publication [22,23]. For the sake of brevity, we reproduce a summarised version here.

### 2.1. Participants and PROCEDURE

The study was carried out among Polish adults who had tested positive for SARS-CoV-2 during an interview within the last year. This was a cross-sectional survey completed online in August 2021. The hyperlink to the Google submission form was put on the authors’ social media pages along with information on the study, its anonymity, and consent to participate, and it was shared among university workers via the researchers’ email. The authors proposed their own classification of the most typical symptoms of COVID-19 because no classification was available in the literature at the time of the study. Of the most common symptoms of SARS-CoV-2 virus infection, the following were selected: febrility cough, myalgia, olfactory and gustatory abnormalities, exhaustion, overall weakness, diarrhoea, dyspnoea or shortness of breath, and cephalalgy [24]. Symptoms were classified as follows: severe, fully symptomatic, oligosymptomatic, and asymptomatic. Fully symptomatic COVID-19 was interpreted as the existence of at least 6 of the 10 prevalent symptoms. The occurrence of up to 3 of the 10 most leading manifestations (in addition to respiratory distress or dyspnoea) was considered oligosymptomatic, whereas no symptoms were considered asymptomatic. Hospitalization due to COVID-19 was considered severe. The study’s inclusion requirements were an age of at least 18 years, consent to participate in the trial, and a reported positive SARS-CoV-2 test within the previous year. This research was carried out in accordance with the principles of the Helsinki Declaration and was granted permission by the Senate Committee on Ethics in Scientific Research of the University of Medical Sciences in Bialystok, KB/162/2020/2021.

### 2.2. Methods of Assessing the Level of Fatigue and PA

#### 2.2.1. Modified Fatigue Impact Scale (MFIS)

The authors assessed the level of fatigue using the MFIS in Polish, which is divided into three parts, F-1 (physical functioning), F-2 (cognitive functioning), F-3 (psychosocial functioning), and includes questions about the patient’s subjective feelings in the last four weeks [25]. Depending on the answers in each part of the questionnaire, a patient can achieve a score between 21 and 105 with a higher score indicating a greater impact of fatigue on the patient’s functioning. The Cronbach’s alpha value given in the publications is >0.7 [26,27].

#### 2.2.2. International Physical Activity Questionnaire (IPAQ)

The study used the short version of the IPAQ whose purpose is to assess and measure PA. The questionnaire consisted of 7 questions on types of daily PA [28], such as during work, home and neighbourhood, and leisure time spent in other physical activities. The PA-assessment methods collected information from the respondents, such as the time spent walking, sitting, and engaging in vigorous and moderate PA [28]. The questionnaire assessed activities that lasted continuously for at least 10 min. All activities are reported in MET-min/week [29]. Based on the results of the IPAQ, the respondents can be classified according to their PA level. A distinction is made between high level—if the respondent, for example, exercises intensively on 3 or more days, i.e., a total of at least 1500 MET-min/week; average level—if the respondent, for example, exercises intensively on 3 or more days, lasting no less than 20 min per day; and low level—if the respondent, for example, does not show any type of PA [28]. The Cronbach’s alpha value given in the publications is >0.7 [29,30].

### 2.3. Statistical Methods

The Kruskal–Wallis test was used to examine the significance of the differences between groups; this statistic and test were chosen due to the significant asymmetry of the measures considered. The correlation between age and measures of fatigue and PA was examined using the Spearman rank correlation coefficient. A significance level of *p* < 0.05 (*) was established for all statistical analyses, but additionally denoted results for *p* < 0.01 (**) and *p* < 0.001 (***) were established for all statistical analyses. Statistica v.13 software (TIBCO Software Inc., Palo Alto, CA, USA (2017)) was used for the statistical analysis.

## 3. Results

### 3.1. General Characteristics of the Respondents

Questionnaires received from 204 respondents (122 females and 82 males) were included in the study (Table 1).

Among those surveyed, 46.6% had been ill with COVID-19 more than 6 months before the date of survey completion. The least number of people, 13.7%, had been diagnosed with SARS-CoV-2 virus infection 1–2 months earlier. The remaining 39.7% had become ill between 3 and 6 months after diagnosis.

### 3.2. PA Level According to the IPAQ Questionnaire

Based on the respondents’ answers to the short version of the IPAQ questionnaire, three submeasures and a summary measure of PA were obtained (Table 2).

The level of PA was also divided into categories: high, medium, and low (Table 3). The fewest respondents reported low activity (approximately 26%), while most reported high activity (approximately 41%). It should be noted, however, that the median scores are much lower, meaning that most individuals have low activity levels, and the average is inflated by the few individuals with high or very high activity levels. It is notable that there were people (8 in total) with zero level of any PA.

### 3.3. Level of Fatigue According to the MFIS Scale

MFIS scale scores are presented as values for three domains: physical fatigue, cognitive fatigue, psychosocial fatigue, and general fatigue. Each domain corresponds to a different number of component questions, so the scores on these measures cannot be compared. The distribution of MFIS measures is presented in a summary of descriptive statistics (Table 4). The MFIS questionnaire was completed by 189 subjects. Considering the range of possible scores (e.g., for the total measure of 84 points), it can be concluded that the level of fatigue in the study group was not high—on average, the MFIS total measure is 77, and the median is 17.

### 3.4. Fatigue and PA Levels and Selected Factors

The authors examined whether the mode of passage of COVID-19 affected fatigue and PA levels (Table 5). There were statistically significant distinctions in all parameters with the best results in those who had no symptoms of SARS-CoV-2 infection, while those with moderate symptoms had lower mental and physical health, and the poorest results were in those who were totally symptomatic.

In contrast, the time since diagnosis of infection was not very significantly associated with most measures (Table 6). The effect of the time since diagnosis is more pronounced only for PA, although the direction of the association here is somewhat surprising—the further back the illness, the lower the level of PA.

There are also very large differences in the measures of psychophysical fitness between men and women in the study group (Table 7).

An analysis of the association between age and fatigue and levels of PA was performed separately for the female and male groups (Table 8). Statistically significant correlations between fatigue and PA and age were found in the male group. The higher the age, the higher the fatigue scores and the lower the PA. In the female group, statistically significant but much weaker correlations are less frequent.

### 3.5. PA and Fatigue

An analysis of the correlation between the level of PA and the overall fatigue index was performed (Table 9). Since the activity level differs between men and women, the correlation analysis was broken down by gender. The results are quite interesting: first, negative correlations are shown (higher fatigue scores are negatively correlated with the level of PA), and second, the correlations are much stronger for men.

## 4. Discussion

The study presented here showed that the majority of people in the study group reported low and average levels of physical activity and, at the same time, not very high levels of fatigue. Fatigue was not high in the study group—on average, the MFIS total score was 25% of the maximum score. A 2021 systematic review examined the level of fatigue associated with SARS-CoV-2 infection at different time intervals with fatigue rates ranging from 9 to 42% at 8 to 11 weeks after the onset of COVID-19 symptoms and approximately 32% at 28 weeks after the first symptoms [31]. There are also reports in the literature that fatigue affected 87% of individuals three months after COVID-19 [32]. However, the differences between populations, the study methods used, and the timing relative to the acute phase of COVID-19 sometimes mean that the degree of fatigue in the different studies cannot be compared.

Total PA levels declined considerably in all age categories between the period immediately preceding and during the COVID-19 pandemic [15]. The authors’ own study showed that the fewest people presented low activity (approximately 26%), while the most people presented high activity (approximately 41%). The rest presented medium activity (33%). However, the authors noted much higher median values, which means that most people present a low level of activity, and the average is inflated by the few people with a high or very high level of PA.

There are statistically significant differences in all measures of fatigue and PA with the best results in those with asymptomatic SARS-CoV-2 infection, a poorer condition in those displaying minor symptoms, and the worst condition in those who showed full symptoms of the disease. However, because of the cross-sectional character of the research, the cause-effect relationships cannot be demonstrated. The authors hypothesize that confounding factors, such as chronic comorbidities or an advanced age of the patients, may have also influenced these results. At the same time, these results are consistent with data from the literature, where potential risk factors for fatigue after SARS-CoV-2 infection were more severe clinical conditions in the acute phase of COVID-19 [5].

The time since diagnosis of infection is not very clearly associated with most measures. Only for PA is the effect more pronounced, although the direction of the relationship here is somewhat surprising—the further back the disease, the lower the PA (the authors consider that this could be related to confounding factors). Regarding the extent of fatigue, this is consistent with some studies in the literature that have found no relationship with the time since symptom onset [33]. However, this is in contrast to other studies. These differences may be related to the severity of the condition or the duration of the follow-up period, for example [34].

In our research, there are considerable differences in fatigue and PA levels between men and women. Part of this is justified—for example, in the IPAQ measures—but part of it is undoubtedly attributable to the enormous age difference between both sexes. According to the findings of this study, women are more prone to become weary at lower levels of PA. The increased fatigue found in women in our study is consistent with previous results that indicated female sex is a potential contributory cause for fatigue following COVID-19 [5,8,34].

There are several theories on the potential reasons for the higher prevalence of fatigue in women [8]. These include greater exposure during the pandemic to psychiatric disorders being predictors of fatigue [35], direct effects of the SARS-CoV-2 virus on skeletal muscles [36], and changes in neurotransmitter levels [37].

The authors’ findings on PA levels in women are congruent with those of other scientists who found that women showed a higher proportion of low levels of PA than men who primarily reported a higher proportion of robust levels of PA [8]. Previous research has also found that women are less likely to participate in activities that can be performed individually [38,39].

Males had statistically significant connections between physical fitness and age. The older the respondent was, the lower the physical exertion scores were. The absence or weaker strength of the correlation in the female group could be due to the fact that there are relatively few subjects in the youngest age group who could be characterised by higher levels of PA and better health and mental status. Nevertheless, age influences the assessment of physical fitness in people after COVID-19, as it does on the risk of fatigue [5].

PA may play a crucial role in influencing the severity of weariness [8]. In the available meta-analyses, the majority of patients with a history of fatigue associated with COVID-19 reported the disappearance of symptoms after rehabilitation treatment or increased levels of PA [40,41]. At the same time, fatigue is one of the strongest predictors of decreases in fitness and PA, as shown by observational studies [42,43]. When analysing the correlation between the level of PA and the overall fatigue index, the authors obtained interesting results. First, negative correlations were observed, which is understandable since higher fatigue levels interact negatively with PA levels. Second, much stronger correlations were observed in men. However, because PA can affect fatigue and vice versa, the findings of this study did not establish a causative association.

The merits of the presented survey were the easy accessibility to the studied group, the use of standardised and approved tools, the low cost of the conducted research, and an increase in knowledge about fatigue and PA in people after COVID-19 in Poland. The study presented here can help plan and design fatigue-prevention programs in post COVID-19 individuals, as it highlights the need to incorporate elements that promote physical activity. It also draws attention to the burden of fatigue in women and the elderly after COVID-19, so these groups should be monitored, especially during the course of a long COVID-19. Unfortunately, the poll has certain shortcomings as well: the survey group’s small size and the subjectivity of the responses. In addition, the study was influenced by confounding factors. Since this is a study based on observation, it is impossible to establish that fatigue and PA are causally related to the severity of COVID-19 and the time since the diagnosis of COVID-19 infection. This could mean that, for example, in patients with concomitant diseases, daily physical activities may be difficult because of their health status, which may also affect the feeling of fatigue. In addition, this was an online-only survey conducted via a link, which restricted access to the questionnaire for those with poor internet access. As a result, the study should be performed on a larger number of people rather than via the internet, and the obtained variables should be assessed using more objective tools (e.g., accelerometry, GPS).

## 5. Conclusions

The high incidence of weariness with even mild COVID-19 instances can carry significant implications for entire populations. This study showed that most people in the study group reported low and average levels of PA and, simultaneously, not high levels of fatigue. The best results were reported by those who were mildly affected with COVID-19. The time since diagnosis (as opposed to female sex and advanced age) was not very clearly associated with most measures (except PA level). Higher levels of fatigue (mainly observed in women) interacted negatively with PA levels. These results show that there are gender-related differences in the level of fatigue and PA in patients after COVID-19 in Poland. Considering that PA may play an important role in regulating the severity of fatigue, it should be increased, especially in patients after COVID-19. Further studies are necessary to explore the mechanism of tiredness and PA variations.

## Figures and Tables

**Table 1:** Sex and age of respondents.

Age (Years)	Sex		Total
	Woman	Man	
<26	15	35	50
26–35	29	20	49
36–45	35	14	49
46–55	39	7	46
>55	4	6	10
Total	122	82	204

**Table 2:** Partial measures of PA.

IPAQ	$\overset{¯}{x}$	Me	*s*	Min	Max
Intense effort	1549	960	1698	0	6160
Moderate effort	798	480	849	0	3080
Walking	776	495	693	0	2541
Total effort	3123	2098	3006	0	11,550

IPAQ—International Physical Activity Questionnaire.

**Table 3:** Summary measures of PA.

Level of PA	Percentage of People
low	26%
average	33%
high	41%

**Table 4:** Distribution of MFIS measures.

MFIS	$\overset{¯}{x}$	Me	*s*	Min	Max
physical functioning	8.4	6	8.8	0	33
cognitive functioning	10.3	10	10.0	0	37
psychosocial functioning	1.9	1	2.1	0	8
holistic functioning	20.6	17	20.3	0	77

MFIS—Modified Fatigue Impact Scale.

**Table 5:** Mode of passage of infection versus fatigue and PA.

MFIS IPAQ	Mode of Transmission of Infection						*p*
	Asymptomatic (*N* = 54)		Oligosymptomatic (*N* = 99)		Fully Symptomatic (*N* = 51)		
	Mean	Median	Mean	Median	Mean	Median	
F-1 (physical functioning)	1.6	0	7.3	6	18.0	18	0.0000 ***
F-2 (cognitive functioning)	3.3	0	9.3	9	19.9	20	0.0000 ***
F-3 (psychosocial functioning)	0.4	0	1.8	1	3.8	4	0.0000 ***
MFIS (total functioning)	5.3	0	18.4	17	41.7	42	0.0000 ***
IPAQ—H	2890	2880	1284	720	671	320	0.0000 ***
IPAQ—M	1397	1440	678	360	409	160	0.0000 ***
IPAQ—L	1167	1188	730	462	462	264	0.0000 ***
IPAQ—total	5454	6134	2691	1937	1542	650	0.0000 ***

*p*—test probability value calculated using Kruskal–Wallis test; *p* < 0.001 (***); MFIS—Modified Fatigue Impact Scale; IPAQ—International Physical Activity Questionnaire.

**Table 6:** Time since COVID-19 diagnosis versus fatigue and PA.

MFIS IPAQ	Time Since Diagnosis of COVID-19 Infection								*p*
	1–2 Months. (*N* = 28)		3–4 Months. (*N* = 47)		5–6 Months. (*N* = 34)		>6 Months. (*N* = 95)		
	Mean	Me	Mean	Me	Mean	Me	Mean	Me	
F-1 (physical functioning)	7.4	0	7.4	4	10.2	8.5	8.6	9	0.1287
F-2 (cognitive functioning)	8.8	0	8.2	1	12.2	10.5	11.1	11	0.0469 *
F-3 (psychosocial functioning)	1.7	0	1.7	0	2,1	2	2.0	2	0.3113
MFIS (total functioning)	18.0	0	17.3	7	24.4	21	21.7	21	0.0485 *
Intense effort	2218	1240	2116	2000	1421	720	1083	480	0.0004 ***
Moderate effort	1020	700	1097	1060	783	480	575	160	0.0024 **
Walking	910	792	978	908	856	693	599	330	0.0032 **
Total effort	4149	3350	4192	4025	3060	2102	2257	1310	0.0019 **

*p*—test probability values calculated using Kruskal–Wallis test; *p* < 0.05 (*); *p* < 0.01 (**); *p* < 0.001 (***); MFIS—Modified Fatigue Impact Scale; IPAQ—International Physical Activity Questionnaire.

**Table 7:** Gender and fatigue and PA.

MFIS IPAQ	Gender				*p*
	Women		Men		
	Average	Median	Average	Median	
F-1 (physical functioning)	10.5	9	5.5	0	0.0000 ***
F-2 (cognitive functioning)	12.8	12	6.8	0	0.0000 ***
F-3 (psychosocial functioning)	2.4	2	1.3	0	0.0000 ***
MFIS (total functioning)	25.6	22	13.5	0	0.0000 ***
Intense effort	812	320	2510	2800	0.0000 ***
Moderate effort	457	160	1243	1200	0.0000 ***
Walking	588	330	1021	1155	0.0000 ***
Total effort	1857	1011	4774	5188	0.0000 ***

*p*—test probability value calculated using Mann–Whitney test; *p* < 0.001 (***); MFIS—Modified Fatigue Impact Scale; IPAQ—International Physical Activity Questionnaire.

**Table 8:** Age versus fatigue and PA.

MFIS IPAQ	Gender	
	Women	Men
	Age	
F-1 (physical functioning)	0.23 (*p* = 0.0142 *)	0.60 (*p* = 0.0000 ***)
F-2 (cognitive functioning)	0.05 (*p* = 0.6098)	0.55 (*p* = 0.0000 ***)
F-3 (psychosocial functioning)	0.14 (*p* = 0.1485)	0.58 (*p* = 0.0000 ***)
MFIS (total functioning)	0.14 (*p* = 0.1389)	0.56 (*p* = 0.0000 ***)
IE	0.02 (*p* = 0.8037)	−0.59 (*p* = 0.0000 ***)
ME	0.00 (*p* = 0.9665)	−0.52 (*p* = 0.0000 ***)
W	−0.12 (*p* = 0.2435)	−0.50 (*p* = 0.0000 ***)
TE	−0.04 (*p* = 0.7237)	−0.56 (*p* = 0.0000 ***)

*p* < 0.001 (***), *p* < 0.05 (*); MFIS—Modified Fatigue Impact Scale; IPAQ—International Physical Activity Questionnaire; IE—Intense effort; ME—Moderate effort; W—Walking; TE—Total effort.

**Table 9:** Intercorrelations of PA level with total fatigue index.

IPAQ	Gender	
	Women	Men
	MFIS (Total Functioning)	
IE	−0.28 (*p* = 0.0047 **)	−0.78 (*p* = 0.0000 ***)
ME	−0.24 (*p* = 0.0139 *)	−0.74 (*p* = 0.0000 ***)
W	−0.27 (*p* = 0.0066 **)	−0.78 (*p* = 0.0000 ***)
TE	−0.31 (*p* = 0.0013 **)	−0.77 (*p* = 0.0000 ***)

*p* < 0.05 (*); *p* < 0.01 (**); *p* < 0.001 (***); IPAQ—International Physical Activity Questionnaire; MFIS—Modified Fatigue Impact Scale; IE—Intense effort; ME—Moderate effort; W—Walking; TE—Total effort.

## Data Availability

The data that support the findings of this study are openly available in RepOD at https://doi.org/10.18150/2YITU6 (accessed on 18 August 2022).
